# Efficacy of a plant-based diet on FOLFOX chemotherapy-induced gastrointestinal toxicity in patients with colorectal cancer: study protocol for a multicentre, stratified, randomised controlled trial

**DOI:** 10.1186/s13063-026-09573-y

**Published:** 2026-03-03

**Authors:** Wei Peng, Hongrui Shi, Liang Fan, Xinxin Fan, Yan Xing, Yuzan Peng, Yuling He, Wei Zou, Tong Yang, Xiaoyu Wang, Guo Cheng

**Affiliations:** 1 West China School of Nursing, Sichuan University/Department of Oncology, West China School of Public Health and West China Fourth Hospital, Sichuan University, Chengdu, 610041 China; 2 College of Nursing, Shanxi University of Chinese Medicine, Jinzhong, 030619 China; 3 College of Nursing, Dazhou Vocational College of Chinese Medicine, Dazhou, 635000 China; 4 Laboratory of Molecular Translational Medicine, Center for Translational Medicine, Key Laboratory of Birth Defects and Related Diseases of Women and Children (Sichuan University), Ministry of Education, Maternal & Child Nutrition Center, West China Second University Hospital, Sichuan University, Chengdu, Sichuan People’s Republic of China; 5 Children’s Medicine Key Laboratory of Sichuan Province, Sichuan University, Chengdu, Sichuan P.R. China

**Keywords:** Colorectal cancer, Chemotherapy-induced gastrointestinal toxicity, CIGT, FOLFOX, Plant-based diet, Inflammation, Gut microbiota, Metabolomics

## Abstract

**Background:**

Gastrointestinal toxicity during FOLFOX chemotherapy for colorectal cancer (CRC) is frequent and can impair oral intake, quality of life, and planned chemotherapy delivery. Safe and scalable dietary strategies are needed as adjuncts to standard supportive care. This trial will evaluate whether a structured plant-based dietary strategy reduces chemotherapy-induced gastrointestinal toxicity (CIGT) and improves related biological and patient-centered outcomes.

**Methods:**

This multicentre, stratified, three-arm, parallel-group, assessor-blinded, superiority randomised controlled trial will enrol 114 adults (18–65 years) with pathologically confirmed CRC receiving (or scheduled to receive) FOLFOX chemotherapy at two hospitals in Chengdu, China. Participants will be randomised (1:1:1), stratified by study site and sex, to (1) a structured plant-based dietary strategy, (2) conventional dietary guidance, or (3) conventional dietary guidance plus an oncology complete nutritional formula, for 6 weeks. Assessments will be performed at baseline and at prespecified time points through week 6. Primary outcomes are (i) the participant-level incidence of any prespecified CTCAE gastrointestinal toxicity of grade ≥ 2 during follow-up, (ii) serum inflammatory biomarkers, and (iii) stool-based profiling. Secondary outcomes include dietary intake and diet quality, body composition by bioelectrical impedance analysis, routine clinical laboratory indices, fatigue, anxiety/depression, physical activity, and adverse events. Primary analyses will follow the intention-to-treat principle.

**Discussion:**

This trial will provide evidence on the efficacy and safety of a plant-based dietary strategy to mitigate CIGT during FOLFOX chemotherapy. Integrated clinical, biomarker, and stool multi-omics measures may also support exploration of biological correlates of symptom burden and treatment tolerance.

**Trial registration:**

Chinese Clinical Trial Registry (ChiCTR), ChiCTR2500095215; registered 03 January 2025. https://www.chictr.org.cn/showproj.html?proj=254099.

**Supplementary Information:**

The online version contains supplementary material available at 10.1186/s13063-026-09573-y.

## Protocol version {2}

Protocol version 3.0, Dated 16 January 2026.

## Introduction

### Background and rationale {9a}

Colorectal cancer (CRC) remains a major global health burden, with approximately 1.92 million new cases worldwide in 2022, and China accounting for nearly one-third of the global burden as incidence continues to rise [1, 2]. Systemic chemotherapy is central to the management of CRC, with FOLFOX (oxaliplatin, leucovorin, and 5-fluorouracil) being widely used as a first-line regimen for advanced disease [3]. It can exert direct cytotoxic effects on tumour cells, thereby delaying disease progression and prolonging survival in patients with CRC [3, 4]. However, chemotherapy is frequently accompanied by a spectrum of treatment-related toxicities, including myelosuppression, neurotoxicity, hypersensitivity reactions, fatigue, and chemotherapy-induced gastrointestinal toxicity (CIGT) [5]. Among these, CIGT is one of the most common and clinically consequential toxicities, typically manifesting as nausea, vomiting, anorexia, mucositis, and diarrhoea [6]. These CIGT symptoms can substantially reduce patients’ quality of life, compromise chemotherapy delivery through dose reductions, delays, or discontinuation, and increase healthcare utilisation and supportive medication costs, thereby adding to patients’ financial burden [6, 7].

At present, the management of CIGT relies mainly on pharmacological prophylaxis and symptom-directed supportive care [8]. Although pharmacotherapy can relieve associated symptoms, its high costs and potential adverse effects may limit long-term use and broad implementation in routine clinical practice [9]. This highlights a clear need for safe, patient-acceptable, and scalable non-pharmacological strategies to complement standard care. Among non-pharmacological options, dietary intervention represents a pragmatic adjunct to standard care, given its favourable safety profile, feasibility, potential cost-effectiveness, and high patient acceptability [10].

Plant-based diets are characterised by a predominance of plant-derived foods and a reduced intake of animal-derived foods, particularly red and processed meat [11]. Higher intakes of dietary fibre, polyphenols, and antioxidant nutrients have been proposed to modulate inflammatory signalling, oxidative stress, and intestinal barrier function, processes implicated in the development and persistence of CIGT [12, 13]. Studies have shown that higher adherence to healthful plant-based dietary patterns is associated with lower systemic inflammation, whereas unhealthful patterns show the opposite association [14]. Moreover, a randomised controlled trial reported that a vegetable-based diet with controlled fibre intake reduced the incidence of cancer treatment-related diarrhoea [15]. Nevertheless, despite evidence linking diet to inflammatory status and treatment-related gastrointestinal outcomes, robust evidence remains limited regarding whether plant-based dietary interventions can mitigate CIGT in patients with CRC during active chemotherapy.

### Explanation for the choice of comparator {9b}

The comparators were selected to reflect routine nutritional management during FOLFOX chemotherapy and to enable an interpretable assessment of the incremental effect of a plant-based dietary intervention. The conventional dietary intervention comparator represents standard supportive dietary guidance commonly provided in oncology care and therefore offers a clinically relevant benchmark. The comparator of conventional dietary intervention supplemented with an Oncology Complete Nutritional Formula was included because comprehensive oral nutritional formulas are frequently used when chemotherapy-related gastrointestinal symptoms compromise appetite or oral intake. Including this arm allows the trial to distinguish effects attributable to a structured plant-based dietary pattern from those related to nutritional supplementation alone.

## Objectives {10}

This trial aims to evaluate the efficacy and safety of a plant-based dietary intervention for mitigating CIGT in patients with CRC receiving FOLFOX chemotherapy, compared with a conventional dietary intervention and a conventional dietary intervention supplemented with an Oncology Complete Nutritional Formula.

Primary objective: To compare changes in gastrointestinal symptom burden, serum inflammatory biomarkers, and gut microbiota composition and structure from baseline to week 6 among the three study groups.

Secondary objectives: To compare, from baseline to week 6, dietary intake and diet quality, emotional status, fatigue, nutritional status, and laboratory measures (blood counts and serum nutritional, metabolic, immunological indicators, and tumour markers) across groups.

Safety objective: To assess safety by comparing intervention-related adverse events (including food allergy, hypoglycaemia, and unintended weight loss) across groups.

## Methods: patient and public involvement, and trial design

### Patient and public involvement {11}

Patients and the public were not involved in the design of the study, including the formulation of the research question, selection of outcomes, or development of the intervention and recruitment strategy. Patients and the public will not be involved in the conduct of the trial (e.g., data collection or study management). During the trial, participant feedback on the acceptability and feasibility of the dietary interventions will be collected to inform refinement of the intervention materials and implementation procedures. The study results will be disseminated to participants in a plain-language summary and, where appropriate, through institutional communication channels.

### Trial design {12}

**Fig. 1:**
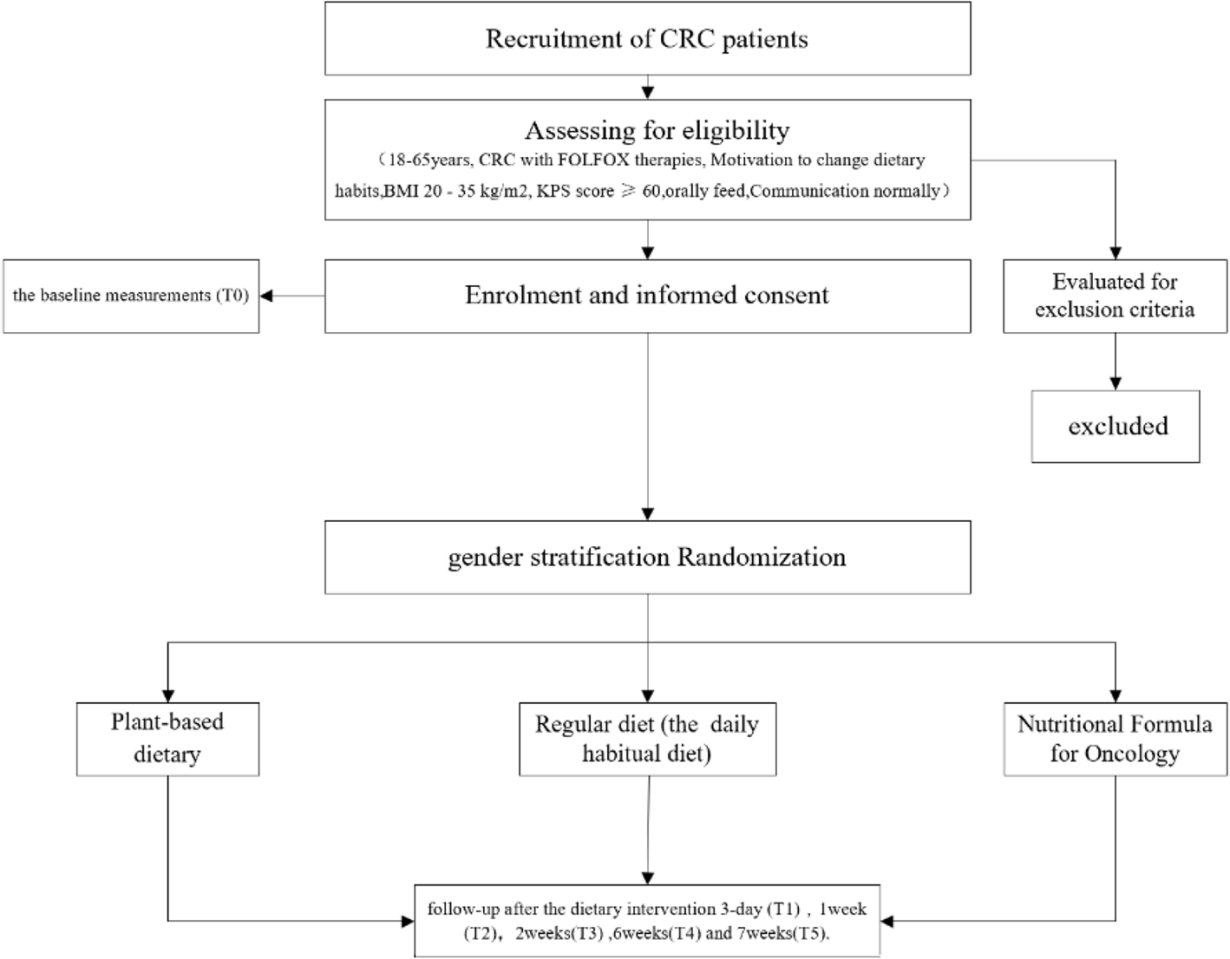
Flow diagram of the study design

The trial is designed with a superiority framework and an allocation ratio of 1:1:1. Participants will be randomised to receive a plant-based dietary intervention, a conventional dietary intervention, or a conventional dietary intervention supplemented with an Oncology Complete Nutritional Formula for a 6-week intervention period. The trial protocol is reported in accordance with the Standard Protocol Items: Recommendations for Interventional Trials (SPIRIT) guidelines [16]. The study flow is illustrated in Fig. 1.

## Methods: participants, interventions and outcomes

### Trial setting {13}

This study will be conducted in Chengdu, China, at two hospital sites: West China Fourth Hospital, Sichuan University, and The Seventh People’s Hospital of Chengdu. Participants will be recruited from the oncology departments of the participating hospitals, where FOLFOX chemotherapy is routinely delivered. The trial will be implemented across inpatient chemotherapy wards and affiliated outpatient services that provide standard supportive care for patients with CRC.

## Characteristics of the people who are needed for the trial

**Table:** 

Characteristic	The people we would expect to see included
Age	Adults 18–65 years with CRC receiving FOLFOX; we expect most participants to be middle-aged/older adults within this range. Age will be recorded at baseline and summarised by group (mean [standard deviation] or median [IQR])
Sex	Both male and female participants are expected. Sex will be extracted from the medical record at baseline and reported by group (n, %)
Gender	Not applicable (sex recorded as male/female from medical records)
Race, ethnicity and ancestry	We expect the majority of participants to be Han Chinese, given the regional population structure and the hospitals’ catchment area; ethnic minority categories will be recorded if self-reported/available and reported descriptively by group
Socioeconomic status	No restrictions are applied. Given the setting (two tertiary hospitals), a broad range is expected. We will collect feasible socioeconomic status indicators (e.g., education level and health insurance type; optionally employment status) and report distributions by group
Geographic location	Recruitment is from two hospitals in Chengdu, Sichuan, China; therefore, most participants are expected to reside in Chengdu and surrounding areas, with a predominance of urban residents. Residence (city/county; urban/rural) will be recorded at baseline and reported by group, acknowledging limits to generalisability beyond this region
Other characteristics relevant to the trial	We anticipate variation in clinical features relevant to tolerability (e.g., disease stage, ostomy status, baseline nutritional risk). These will be collected from medical records and reported descriptively by group

### Eligibility criteria for participants {14a}

#### Inclusion criteria

Participants will be eligible if they meet all of the following criteria:

- Aged 18–65 years.
- Histologically confirmed CRC and scheduled to receive FOLFOX chemotherapy, with an anticipated total chemotherapy duration of ≥ 2 months.
- BMI 20–35 kg/m^2^ and KPS ≥ 60.
- Able to communicate effectively and complete study procedures and questionnaires.
- Able to consume an oral diet and not requiring enteral or parenteral nutrition support at baseline.
- Self-reported motivation to change dietary habits rated as “very motivated” or “motivated”.
- Willing to participate voluntarily and provide written informed consent.

#### Exclusion criteria

Participants will be excluded if they meet any of the following criteria:

- Participation in another randomised controlled trial that could conflict with this study.
- End-stage disease with an estimated life expectancy of < 6 months.
- Inability to understand or adhere to the intervention due to altered mental status, severe cognitive impairment, or severe visual, hearing, or language impairment.
- Unable to complete follow-up procedures (e.g., no reliable contact information for the participant/caregiver).
- Medical or clinical conditions that would preclude adherence to the dietary intervention or safe participation (e.g., pathological eating disorder, severe malnutrition, morbid obesity, or other conditions judged by the investigator).
- Food allergies or intolerances to key components of the study diet, or other conditions that prevent consumption of the assigned diet.
- Use within the past 4 weeks of antibiotics, probiotics, multivitamins, or dietary supplements (including fish oil, omega-3 fatty acids, herbal supplements, calcium, or vitamin D).
- Use of nonsteroidal anti-inflammatory drugs (NSAIDs), antihistamines, or immunosuppressive medications, or inability to refrain from these substances during protocol-specified peri-sampling windows (as defined in the protocol).
- Heavy smoking (> 20 cigarettes/day) or excessive alcohol consumption (> 2 standard drinks/day), or unwillingness to abstain from smoking and alcohol for the duration of the study.
- Significant comorbidities such as autoimmune disease, haematological disease, cardiovascular disease, or active infection, which in the investigator’s judgement would compromise safety or study integrity.

#### Criteria for withdrawal/discontinuation

Participants may be withdrawn or discontinue the intervention if:

- The participant withdraws consent.
- The participant’s clinical condition deteriorates and continued participation is no longer appropriate.
- A serious adverse event occurs (e.g., hypoglycaemia, weight loss > 2 kg/week, food allergy).
- Adherence remains consistently poor.
- Scheduled visits are missed for unforeseen reasons.

### Eligibility criteria for sites and those delivering interventions {14b}

The trial will be conducted at two hospital sites: West China Fourth Hospital, Sichuan University and The Seventh People’s Hospital of Chengdu, both of which are tertiary hospitals with established oncology departments. These sites must have the necessary infrastructure to administer FOLFOX chemotherapy as part of routine care for CRC patients and be equipped to deliver dietary interventions according to the trial protocol. Sites must also obtain local ethical approval and adhere to the study’s protocol, ensuring compliance with ethical standards and local regulations.

Individuals delivering the dietary interventions must have relevant qualifications and experience in oncology nutrition. They should be trained in providing plant-based dietary interventions and be capable of delivering the intervention as outlined in the protocol. All intervention providers will undergo study-specific training to ensure consistency in their approach, which includes dietary guidelines, participant engagement, and monitoring adherence. These individuals must be able to effectively communicate with participants, support adherence, and manage any questions or concerns related to the dietary intervention.

### Who will take informed consent? {32a}

Informed consent will be obtained by the Principal Investigator (WP) prior to any study-specific procedures. The participants will be fully informed about the study’s purpose, procedures, and potential risks. After written informed consent, participants will be randomised (1:1:1) using stratified block randomisation by study site and sex.

### Additional consent provisions for collection and use of participant data and biological specimens {32b}

In addition to the primary consent for participation in the study, participants will be asked for additional consent to collect and use their biological specimens (e.g., blood, stool samples) and data (e.g., inflammatory biomarkers, metabolic indicators) for ancillary studies. These specimens and data will be used solely for the purposes of this study and related research approved by the institutional ethics committee.

Participants will be informed about the storage, handling, and future use of their samples. Biological specimens will be stored at designated facilities under appropriate conditions (e.g., −80 °C storage for biological samples). Biological specimens and datasets will be labelled with unique study Identifiers (IDs) only. The linkage file between personal identifiers and study IDs will be stored separately with restricted access. Samples will be processed and analysed in a de-identified manner, and only aggregated results will be reported. Participants may withdraw consent for future use of stored specimens at any time, without affecting their participation in the main trial.

## Intervention and comparator

### Intervention and comparator description {15a}

This is a multicentre, stratified, randomised controlled trial comparing three different dietary interventions for CRC patients undergoing FOLFOX chemotherapy: plant-based dietary intervention, conventional diet, and oncology nutritional formula. Each group will receive a 6-week dietary intervention during their chemotherapy cycle.

To minimise non-specific (attention/support) effects across groups, the frequency and mode of participant contacts will be standardised across all three arms. All participants will receive: (i) a structured face-to-face education session at baseline during chemotherapy admission (T0), and (ii) daily remote follow-up throughout the 6-week intervention period (telephone/WeChat) delivered according to a prespecified checklist, primarily to record daily dietary intake and monitor symptoms/adverse events. Brief reinforcement will also be provided during inpatient chemotherapy visits when applicable. Unscheduled contacts for symptom management are permitted in all groups and will be documented. Daily contacts are primarily for data capture and safety monitoring; no additional individualized dietary counselling beyond the allocated programme will be provided unless clinically indicated.

The plant-based diet will be based on the latest evidence for nutritional and dietary interventions in managing CIGT. The diet will include fruits, vegetables, whole grains, nuts, and anti-inflammatory beverages such as green tea, soya milk, and peanut-legume juice. It will emphasize reducing red and processed meat intake and limit fish consumption. Participants will also be encouraged to follow the Dietary Guidelines for Chinese Residents [17], which include recommendations on fluid intake (1,500–1,700 ml/d), limited salt (< 5 g/d), and oil (25–30 g/d). Weekly food supplies, printed materials, and cooking videos focused on plant-based nutrition will be provided to support adherence. Quantitative targets (intervention “dose”) were prespecified to operationalize the plant-based dietary strategy: (i) vegetables and fruits ≥ 500 g/d; (ii) whole grains 70–90 g/d and legumes ≥ 1 serving/day on most days; and (iii) red and processed meat ≤ 500 g/week (fish, if consumed, limited to ≤ 300–450 g/week). These targets will be reinforced during follow-up and assessed using diet records and Food Frequency Questionnaire (FFQ)-based scoring.

The conventional diet group will receive standard dietary education and care based on the Dietary Guidelines for Chinese Residents [17]. This includes education materials and guidance according to commonly accepted oncology dietary practices. Participants will not receive individualized dietary counseling unless they seek advice regarding chemotherapy-related symptoms.

The oncology nutritional formula group will receive a commercially available oncology complete nutritional formula. The formula contains protein (including essential amino acids), fat (including essential fatty acids), vitamins (A, C, and E), and minerals. Participants will dissolve one pouch (20 g) in 100 mL of warm water and consume it twice daily for 6 weeks (target dose: 2 pouches/day). Adherence will be monitored using daily intake records and, where feasible, sachet counts/returns. If intolerance occurs (e.g., nausea, bloating, diarrhoea, or poor palatability), intake may be temporarily reduced to one pouch/day or divided into smaller portions, with re-titration to the target dose as tolerated; all dose modifications and reasons will be documented.

The dietary interventions will be delivered for 6 weeks, with participants receiving the intervention during their chemotherapy admission and home-based periods. The interventions will be supported through face-to-face visits, phone calls, and online communication (e.g., via WeChat). Dietitians, oncology nurses, and oncologists will support patients to ensure adherence to the protocol.

### Criteria for discontinuing or modifying allocated intervention/comparator {15b}

The allocated dietary intervention may be discontinued or modified for an individual participant if clinically indicated, including at the participant’s request, on the occurrence of a serious adverse event judged to be related to the intervention (e.g., food allergy or intolerance), or if the participant develops a medical condition that precludes safe continuation of the assigned diet (e.g., inability to maintain oral intake or a requirement for enteral/parenteral nutrition). Thereafter, the participant will receive standard clinical nutritional care as determined by the treating team, and the reason for discontinuation or modification will be documented. Discontinuation or modification of the allocated intervention will not automatically result in withdrawal from study follow-up; outcome assessments will be continued whenever possible.

### Strategies to improve adherence to intervention/comparator {15c}

Adherence will be promoted through structured education and ongoing support delivered by oncology nutrition specialists. At enrollment, participants will receive standardised written materials aligned with their allocated dietary programme, and key dietary targets will be explained to the participant and, where appropriate, a family caregiver. During the 6-week period, daily telephone/WeChat contacts will be used to record dietary intake (diet diary capture), reinforce the allocated strategy, and identify barriers (e.g., nausea, anorexia, oral mucositis, diarrhoea) that may affect intake. A prespecified checklist will be used to standardise contacts across groups. Participants will be asked to complete dietary records at prespecified time points; these records will be reviewed by the study team to assess adherence, provide feedback, and document deviations. For participants allocated to the oncology nutritional formula group, intake will be monitored by recording the number of servings consumed, and unused sachets will be returned or counted when feasible. For participants allocated to the plant-based diet programme, adherence will be assessed against the predefined dietary targets (e.g., plant food intake and restriction of red/processed meat) using dietary records and the plant-based diet index. The predefined targets include vegetables and fruits ≥ 500 g/d, whole grains 70–90 g/d with legumes consumed on most days, and red/processed meat ≤ 500 g/week (fish ≤ 300–450 g/week). Online group interactions will be used to maintain engagement, answer questions, and provide practical guidance (e.g., meal planning and preparation).

### Concomitant care permitted or prohibited during the trial {15d}

During the trial, participants will be permitted to continue their standard pharmacological treatments for CRC, including chemotherapy (e.g., FOLFOX), as prescribed by their oncologist. Routine medical care, including visits to the oncology clinic, will also be maintained according to the standard practice of the participating hospitals. Pain relief medications and other supportive treatments, such as antiemetics or antidiarrheals, will be allowed if necessary for managing chemotherapy-related symptoms.

To maintain the integrity of the study, the use of dietary supplements (including fish oil, omega-3 fatty acids, multivitamins, calcium, vitamin D, or herbal supplements) outside of the study interventions will be prohibited. The use of probiotics will also be restricted to avoid potential confounding effects on gut microbiota and gastrointestinal symptoms. Furthermore, participants will not be permitted to follow any other dietary interventions, such as weight loss programmes or therapeutic diets, unless recommended by their treating physician for medical reasons. The use of NSAIDs and immunosuppressive medications will be restricted, except for cases where these are prescribed for managing medical conditions unrelated to the study. Any changes in concomitant care, including medication use, will be documented in the participant’s case report form to ensure the accuracy of the study outcomes.

### Ancillary and post-trial care {34}

Participants will continue to receive all routine clinical care for CRC, including standard supportive management of treatment-related symptoms, throughout the trial, and any medical issues arising during participation will be managed or referred according to usual practice at the participating hospitals. After the final follow-up assessment, participants will return to routine care under their treating oncology team, with ongoing nutritional advice and supportive care provided as clinically indicated. Any trial-related harm will be managed promptly through the established clinical and administrative pathways of the participating hospitals, including the procedures in place for addressing research-related injury and compensation.

### Outcomes {16}

#### Primary outcomes

- Grades of CIGT: CIGT will be assessed using the National Cancer Institute Common Terminology Criteria for Adverse Events (CTCAE) v5.0 [18]. Prespecified gastrointestinal toxicities include nausea, vomiting, anorexia, oral mucositis, and diarrhoea. Assessments will be conducted at baseline (T0), day 3 post-chemotherapy (T1), week 1 (T2), week 3 (T4), and week 5 (T6). The confirmatory clinical endpoint is the participant-level incidence of experiencing any prespecified CTCAE gastrointestinal toxicity of grade ≥ 2 during follow-up. Longitudinal CTCAE grades, time-specific binary indicators (grade ≥ 2 at each time point), and the maximum CTCAE grade across the prespecified toxicities will be analysed and reported as supportive outcomes (reported as n [%] and median [IQR]).
- Serum inflammatory biomarkers: Serum biomarkers, including interleukin-6 (IL-6) and C-reactive protein (CRP), will be measured at the following time points: T0 and week 6 post-chemotherapy (T7). Outcomes will be summarised as concentrations at each time point and change from baseline to week 6 [reported as mean ± standard deviation (SD)].
- Stool-based multi-omics: Stool samples will be collected at T0, T1, T2, T3, and T7 using a standardised kit and stored at − 80 °C until batch processing. Shotgun metagenomic sequencing will be performed to characterise taxonomic composition and metagenome-derived functional profiles [19]. Sequencing reads will undergo prespecified quality control, filtering, and taxonomic/functional profiling using validated pipelines. Untargeted stool metabolomics will be performed in parallel, with pooled quality control (QC) samples, process blanks, and internal standards included and run order randomised. Metabolomics data will be processed using prespecified procedures (peak detection, alignment, QC-based feature filtering, normalisation/transformation, and batch/drift correction), and metabolite annotation will be supported by tandem mass spectrometry (MS/MS) spectral matching against curated libraries. Feature-level and pathway-level analyses will control multiple testing using false discovery rate procedures. Outcomes will be summarised as within-sample diversity, between-sample dissimilarity, and longitudinal changes in the relative abundance/intensity of key taxa, functional features, and metabolite features/pathways (reported as change from T0 at each time point).

#### Secondary outcomes

- Dietary assessment: Dietary assessment will be conducted using a semi-quantitative FFQ and diet records (food diaries). The FFQ will capture usual dietary intake over the preceding week, including consumption frequency and portion size, and will be administered at T0 and T7. FFQ data will be used to derive plant-based diet indices [Plant-Based Diet Index (PDI), healthy Plant-Based Diet Index (hPDI), and unhealthy Plant-Based Diet Index (uPDI)] by applying pre-specified scoring to plant- and animal-food groups, adapted to the Chinese Dietary Guidelines and the study intervention targets [17]. Diet records will prospectively document all foods and beverages consumed over three consecutive days at a randomly selected time during the intervention period (T0-T7). Outcomes will be summarised as index scores at each FFQ time point and change from T0 to T7, and as average daily intake estimates from the 3-day records (reported as mean ± SD).
- Nutrition assessment: Nutritional status will be evaluated using bioelectrical impedance analysis [Bioelectrical Impedance Analysis (BIA)] and routinely collected clinical laboratory tests at prespecified time points. BIA will be performed with the InBody 370 at T0 and T7 by trained study staff using a standardised procedure [20], yielding body weight and body-composition measures (e.g., fat mass, fat-free mass, skeletal muscle mass, and segmental composition in appendicular and trunk compartments). Routine blood tests will be extracted from the hospital laboratory information system at T0, T3, T5, and T7. Prespecified laboratory domains include nutrition-related markers, lipid and glucose profile, liver and renal function indices, immune cell counts, and tumour markers (Table 1). Outcomes will be summarised as values at each time point and as change from T0 (reported as mean ± SD).
- Fatigue: Fatigue will be assessed using the Chinese version of the Piper Fatigue Scale-12 (PFS-12) [21], a 12-item self-report instrument covering four dimensions (behavioural, affective, sensory, and cognitive). Each item is rated on a 0–10 numeric scale, and the total score is calculated as the mean of all 12 items (range 0–10), with higher scores indicating more severe fatigue. Assessments will be completed at T0, T3, and T7. Outcomes will be summarised as (i) the PFS-12 total score at each time point and (ii) change from T0 at each follow-up (reported as mean ± SD).
- Anxiety and depression: Anxiety and depression will be assessed using the Hospital Anxiety and Depression Scale (HADS) [22], a 14-item self-report instrument comprising two 7-item subscales (anxiety and depression). Items are rated on a 4-point scale, yielding subscale scores from 0 to 21, with higher scores indicating greater symptom severity. Assessments will be completed at T0, T3, and T7. Outcomes will be summarised as (i) anxiety and depression subscale scores at each time point and (ii) change from T0 at each follow-up (reported as mean ± SD).
- Physical activity: Physical activity will be assessed using the Chinese short form of the International Physical Activity Questionnaire (IPAQ-C) [23]. The questionnaire captures self-reported walking, moderate-intensity activity, and vigorous-intensity activity over the previous 7 days. Participants will be classified into low, moderate, or high physical activity according to the IPAQ scoring protocol. Assessments will be completed at T0, T3, and T7. Outcomes will be summarised as the proportion of participants in each physical activity category at each time point and the change in category from T0 to each follow-up (reported as n, %).

**Table 1:** Routine blood tests

Category	Blood counts
Nutrition-related markers	TP, ALB, Hb, Fe, Mg, Ca
Liver and renal function indices	ALT, AST, Cr, eGFR
Lipid- and glucose-related markers	TC, TG, LDL-C, HDL-C, glucose
Immune-related cell counts	WBC, neutrophil count, TLC
Tumour markers	CEA, CA19-9

*TP* total protein, *ALB* albumin, *Hb* haemoglobin, *Fe* iron, *Mg* magnesium, *Ca* total calcium, *ALT* alanine aminotransferase, *AST* aspartate aminotransferase, *Cr* creatinine, eGFR estimated glomerular filtration rate, *TC* total cholesterol, *TG* triglycerides, *LDL-C* low-density lipoprotein cholesterol, *HDL-C* high-density lipoprotein cholesterol, *WBC* white blood cell count, *TLC* total lymphocyte count, *CEA* carcinoembryonic antigen, *CA19-9* carbohydrate antigen 19–9

### Harms {17}

Potential harms related to the dietary interventions include food allergy/intolerance, hypoglycaemia, clinically relevant weight loss, headache, and other unexpected symptoms during the intervention period. Adverse events will be actively captured at each scheduled contact from T0 to T7, and participants may report events between contacts via telephone or WeChat. All adverse events will be recorded on a prespecified case report form and graded using CTCAE v5.0 [18]. For each event, onset/resolution dates, CTCAE grade, relatedness to the intervention, actions taken, and outcome will be documented. Serious adverse events and events requiring urgent medical attention will be managed according to routine clinical practice and reported to the ethics committee in a timely manner. Safety data will be reviewed by a designated safety monitor, who may recommend modification or discontinuation of the intervention if unacceptable harms are observed. Harms will be summarised by group as incidence and severity distribution, with particular attention to grade ≥ 2 events, serious adverse events, and events leading to discontinuation.

### Participant timeline {18}

T0 is defined as baseline (before initiation of the allocated dietary intervention and within the chemotherapy admission). Follow-up time points are anchored to the start of the chemotherapy cycle: T1, day 3 post-chemotherapy; T2, week 1; T3, week 2; T4, week 3; T5, week 4; T6, week 5; and T7, week 6. There is no run-in or washout period. After eligibility screening and written informed consent, participants will be randomised (1:1:1) to one of three dietary strategies and followed for 6 weeks across inpatient chemotherapy and the home-based interval period. Assessments will be conducted in person during inpatient visits when feasible and otherwise via scheduled telephone/WeChat contacts according to the timetable below. Table 2 shows the participant timeline.

**Table 2:**
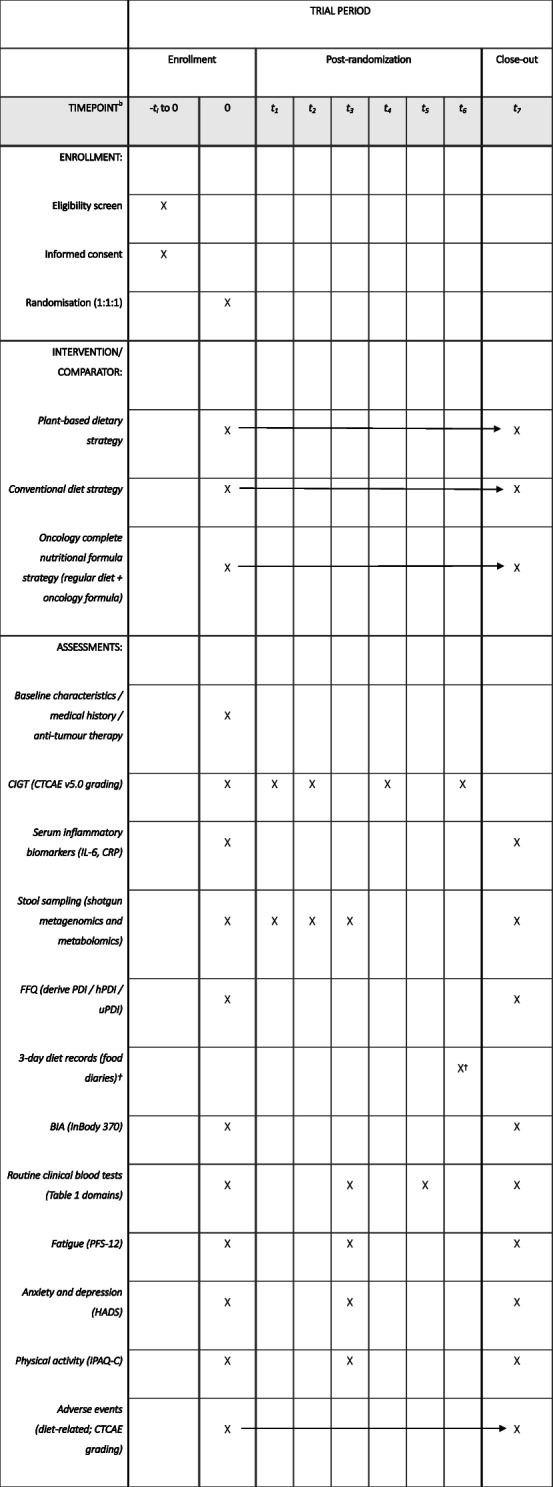
Schedule of enrollment, interventions, and assessments

^†^3-day diet records: The diet record will be completed once at a randomly selected time point during the intervention period (T0-T7)

X indicates discrete time-point activities/assessments

Horizontal arrows indicate procedures delivered continuously over the interval (t0-t7)

### Sample size {19}

Sample size was estimated using the Tests for Two Proportions module in PASS 2025 for the primary endpoint, defined as experiencing any prespecified CTCAE gastrointestinal toxicity of grade ≥ 2. Given two prespecified primary comparisons, a Bonferroni-adjusted two-sided α of 0.025 and 80% power were applied. Based on Souza et al. [24], the assumed proportions were 0.176 in the intervention group and 0.533 in the control group. Under these assumptions, 30 evaluable participants per group are required for each primary comparison. To account for an anticipated 20% loss to follow-up, this was rounded up to 38 participants per arm (total N = 114; 1:1:1).

### Recruitment {20}

Participants will be recruited from two sites in Chengdu, China (West China Fourth Hospital, Sichuan University, and The Seventh People’s Hospital of Chengdu). Recruitment began on 10 January 2026. Potentially eligible patients will be identified through routine inpatient and outpatient oncology services and clinician referrals. Study information will be provided via clinic-based postings (posters/flyers) and the departments’ official online channels (e.g., website and WeChat public account). Interested patients will contact the study team by telephone or WeChat and will complete a brief pre-screening to assess key eligibility criteria, followed by on-site screening. Recruitment will be tracked using screening logs at both sites. If recruitment falls behind target, recruitment efforts will be intensified through clinician reminders, refreshed materials, expanded online dissemination, and adjustments to recruitment coverage and/or the recruitment period.

## Assignment of interventions: randomisation

### Sequence generation: who will generate the sequence {21a}

The random allocation sequence will be generated by XYW using a computer-generated random-number algorithm. The sequence generator will not be involved in participant enrollment or group assignment.

### Sequence generation: type of randomisation {21b}

Participants will be randomly allocated to one of three study arms in a 1:1:1 ratio using restricted randomisation with permuted blocks (variable block sizes). Randomisation will be stratified by study site (two hospitals) and sex (male/female). To reduce predictability, details of block sizes will be stored in a separate document that is not accessible to personnel who enroll participants or assign interventions.

### Allocation concealment mechanism {22}

Allocation will be concealed using sequentially numbered, opaque, sealed envelopes prepared for each stratum (site × sex). Envelopes will be tamper-evident and stored securely at each recruiting site under the custody of an allocation administrator who is not involved in participant enrollment or outcome assessment. The next envelope in sequence will be opened only after participant eligibility is confirmed and baseline assessments have been completed.

### Implementation {23}

Participant enrollment will be undertaken by WZ. Following completion of baseline assessments, group assignment will be implemented by the allocation administrator (a member of the intervention delivery team) by opening the next envelope in sequence. WZ and outcome assessors/data collectors will not have access to the random allocation sequence, block sizes, or allocation records, and will remain blinded to group assignment.

## Assignment of interventions: blinding

### Who will be blinded {24a}

Blinding will be maintained using coded intervention labels (Programme A/B/C) and role separation. Participant-facing materials will use programme codes only. Screening personnel and outcome data collectors will be separate from intervention providers and will not have access to the allocation codebook. Biological samples and datasets will be labelled with study IDs only. Participants will be reminded not to disclose intervention details to blinded staff.

### How will be blinding be achieved {24b}

Blinding will be maintained through role separation and coded intervention labels. The three interventions will be presented as coded nutrition programmes (e.g., Programme A/B/C), and all participant-facing materials will use these codes. Allocation information will be accessible only to the intervention delivery team via an allocation codebook; recruitment staff, assessors, laboratory personnel, and statisticians will not have access. Participants will be reminded not to disclose details of their dietary programme to blinded staff.

### Procedure for unblinding if needed {24c}

Unblinding will be permissible only when knowledge of the assigned intervention is essential for clinical management or participant safety. Any request for unblinding will be authorised by the principal investigator, and the allocation will be revealed by the intervention delivery team using the allocation codebook. The reason, date, personnel involved, and the participant(s) unblinded will be documented. Outcome assessors will remain blinded whenever possible.

## Data collection and management

### Plans for assessment and collection of outcomes {25a}

All participants will provide demographic and baseline information at enrollment. Outcome assessments will be conducted at the prespecified time points shown in Table 2, using standardised case report forms and study-specific Standard Operating Procedures (SOPs). Validated and standardised instruments will be used (e.g., CTCAE; PFS-12, HADS, and IPAQ-C). Informed consent will be obtained by the Principal Investigator (WP) according to IRB-approved procedures. A trained research assistant (XXF) will support participant-facing data collection by providing standardised instructions and assisting completion without coaching responses. Data will be recorded contemporaneously, checked for completeness at the point of collection, and any discrepancies will be clarified against source documents and documented.

### Plans to promote participant retention and complete follow-up {25b}

To promote retention and complete follow-up, participants will receive enrollment education and written materials (brochures) and will be reminded of scheduled assessments via WeChat messages, reminder cards, and telephone calls. Whenever possible, follow-up will be aligned with routine chemotherapy-related contacts, with flexible scheduling within predefined visit windows. Missed visits will trigger repeated contact attempts, and reasons for missed visits, discontinuation, or protocol deviations will be documented. Participants who discontinue or deviate from the allocated dietary intervention will be encouraged to remain in follow-up; every reasonable effort will be made to collect key outcome and safety data at the scheduled time points to support intention-to-treat analyses. If a participant withdraws consent for further data collection, no additional data will be collected, and the withdrawal reason will be recorded if the participant agrees.

### Data management {26}

Trial data will be entered into a de-identified study database using prespecified coding rules and a data dictionary. Questionnaire data will be captured in SoJump using study IDs only [25]; access is role-restricted, and exported datasets will be stored on a secure, access-controlled institutional drive. Data quality will be promoted through predefined range and logic checks for key variables, routine missing-data checks, and query-based correction against source documents (e.g., medical records and laboratory information system outputs). Entered data will be independently reviewed and signed off by two investigators (LF and YX), with oversight by the trial leadership (WP and GC) to ensure consistency and completeness. Access will be password-protected and role-based; the ID–identity linkage file will be stored separately with restricted access. Regular backups will be performed and stored securely. Signed informed consent forms and other paper records will be stored in locked cabinets in secure offices. Only de-identified datasets will be released for statistical analyses, and the final analytic dataset will be accessible only to the designated analysts (HRS and WP). Data will be retained and archived according to applicable regulations and institutional policies.

### Confidentiality {33}

All potential and enrolled participants will be assigned a unique study identification code. This code, rather than personal identifiers, will be used on all study documents and datasets, including case report forms, questionnaires, and biological samples. The master list linking identifiers (e.g., name and contact details) to study codes will be stored separately from research data in a secure, access-restricted location; access to identifiable information will be limited to the Principal Investigator (WP) and authorized key study personnel as required for trial conduct and follow-up. Any data shared for analysis or laboratory processing will use study codes only and will be de-identified before export. No participant-identifiable information will be included in publications, presentations, or shared reports; results will be reported in aggregate. After trial completion, records will be archived securely for the period required by applicable regulations and institutional policies, and personal data will be disposed of securely after the mandatory retention period in accordance with institutional procedures.

## Statistical methods

### Statistical methods for primary and secondary outcomes {27a}

Analyses will be prespecified in the statistical analysis plan (SAP) and conducted using R and/or SPSS. Baseline characteristics will be summarized by group using mean ± SD or median (IQR) for continuous variables and n (%) for categorical variables. Two primary comparisons will be performed: plant-based diet versus conventional diet, and plant-based diet versus conventional diet plus oncology nutritional formula. To control the family-wise type I error across the two primary comparisons, we will use a Bonferroni-adjusted two-sided α of 0.025 for each comparison [26]. Within each comparison, the three co-primary outcomes will be tested using a pre-specified hierarchical gatekeeping procedure in the following order: (1) CIGT, (2) serum inflammatory biomarkers, and (3) stool-based multi-omics. For the confirmatory test of the first co-primary outcome (CIGT), the primary clinical endpoint is the participant-level incidence of experiencing any prespecified CTCAE gastrointestinal toxicity of grade ≥ 2. Testing will proceed to the next outcome only if the preceding outcome is statistically significant at the same two-sided α level; otherwise, subsequent outcomes will be analysed and reported as supportive/exploratory without confirmatory claims.

The confirmatory analysis of the primary clinical endpoint will use logistic regression with adjustment for the stratification factors (site and sex) and other prespecified covariates as applicable. Longitudinal continuous outcomes will be analysed using linear mixed-effects models with fixed effects for group, time, and group-by-time interaction and a participant-level random intercept (and random slope if supported), adjusting for the baseline value of the outcome (when applicable) and the stratification factors. Longitudinal ordinal CTCAE grades and time-specific binary indicators (e.g., grade ≥ 2 at each time point) will be analysed as key secondary/supportive outcomes using mixed-effects ordinal logistic models and mixed-effects logistic regression, respectively, with the same covariate structure.

Microbiome and metabolomics outcomes will be analysed according to prespecified metrics. For metabolomics, multivariate structure will be explored using ordination, and feature-level differences over time will be tested using regression/mixed-effects frameworks with false discovery rate (FDR) control; pathway-level enrichment analyses will be reported as supportive. Community-level differences will be assessed using distance-based methods, and feature-level analyses will apply appropriate regression frameworks with FDR control for multiple testing. Harms events will be summarized by group (incidence, severity, seriousness, and relatedness) and compared using χ^2^/Fisher’s exact tests or regression models as appropriate; serious adverse events and events leading to intervention discontinuation will be described descriptively.

### Who will be included in each analysis {27b}

The primary analysis will follow the intention-to-treat (ITT) principle, including all randomised participants and analysing them in the groups to which they were allocated, regardless of adherence or protocol deviations. A per-protocol sensitivity analysis will be conducted excluding participants with major protocol violations and/or not meeting prespecified adherence criteria. Safety analyses will include all participants who received any component of the assigned intervention/comparator.

### How missing data will be handled in the analysis {27c}

Every effort will be made to minimise missing data through the retention procedures described above. For longitudinal outcomes, mixed-effects models using maximum likelihood estimation will be the primary approach and can accommodate missing outcome data under a missing-at-random assumption. If additional handling is required (e.g., substantial missingness in key secondary outcomes), multiple imputation will be applied using prespecified models incorporating baseline variables, stratification factors, and available outcome history; results will be compared with complete-case analyses as sensitivity checks. For microbiome/metabolomics features, no imputation will be performed for unavailable samples; analyses will be based on available specimens, with missingness patterns summarized and sensitivity analyses performed where appropriate.

### Methods for additional analyses (e.g. subgroup analyses) {27d}

Prespecified subgroup analyses will be exploratory and will assess potential effect modification by site and sex (stratification factors), and other clinically relevant baseline characteristics as prespecified in the SAP. Subgroup effects will be evaluated by adding interaction terms (e.g., group × subgroup and group × time × subgroup where relevant) within the primary modeling framework. Adjusted analyses (e.g., additionally controlling for baseline values and key covariates) will be prespecified and reported alongside unadjusted summaries.

### Interim analyses {28b}

No formal interim analysis for efficacy is planned. Safety data will be monitored continuously by the study team, and any concerns regarding unexpected or unacceptable intervention-related harms will be reviewed by the Principal Investigator, with escalation and reporting to the ethics committee as required. Decisions to pause or terminate the trial for safety reasons will be made by the Principal Investigator in accordance with IRB/ethics requirements.

### Protocol and statistical analysis plan {5}

This protocol will be publicly accessible through publication and trial registration records. The SAP will be finalized before database lock and before unblinding of the analytic dataset, approved by the study leadership, and made available as a supplementary file with the protocol/publication or upon reasonable request from the corresponding authors.

## Oversight and monitoring

### Composition of the coordinating centre and trial steering committee {3d}

Day-to-day trial delivery will be coordinated by a Trial Management Group (TMG) based at the central coordinating centre. The TMG will be responsible for overall trial operations, including site start-up and training, recruitment coordination, intervention delivery oversight, protocol adherence, data management coordination, safety reporting, and preparation of trial reports. The TMG will include the chief/principal investigator(s), a trial coordinator/manager, site investigators from each participating hospital, intervention delivery representatives, a data manager, and a statistician. The TMG will meet regularly (e.g., every 2–4 weeks) and additionally as required to address operational issues. Independent oversight will be provided by a Trial Steering Committee, which will review trial progress, protocol compliance, and participant safety at predefined intervals (e.g., every 6 months) and advise on any major trial decisions; the TSC will convene additional meetings if triggered by safety concerns or major protocol issues.

### Composition of the data monitoring committee, its role and reporting structure {28a}

An independent Data Monitoring Committee (DMC) will oversee participant safety and trial integrity. The DMC will comprise at least three members with relevant expertise (e.g., clinical oncology, nutrition/supportive care, and statistics) who are independent of the sponsor/funder and the study investigators, with no competing interests. The DMC will periodically review accumulating safety data and key trial conduct indicators (e.g., recruitment, retention, protocol deviations) and may request unblinded summaries if necessary for safety evaluation. The DMC will provide written recommendations to the Trial Steering Committee/Chief Investigator regarding continuation, modification, temporary suspension, or early termination of the trial on safety grounds. Any serious safety concerns will be escalated promptly according to the reporting pathway defined in the DMC charter (available from the coordinating centre upon request).

### Frequency and plans for auditing trial conduct {29}

Trial conduct will be monitored using a risk-based approach aligned with Good Clinical Practice. Monitoring activities will include central checks of data completeness/consistency, verification of key documents (e.g., informed consent, eligibility), and review of adverse event reporting. On-site or remote monitoring visits may be conducted periodically (e.g., at least annually per site or more frequently if risk triggers arise), focusing on critical processes such as consent procedures, adherence to eligibility criteria, protocol compliance, source data verification for primary outcomes and serious adverse events, and data security practices. Audit findings will be documented, corrective actions will be agreed with the site team, and follow-up will be performed to ensure resolution.

### Protocol amendments {31}

Any important protocol modifications (e.g., changes to eligibility criteria, intervention procedures, outcomes, or analyses) will be documented as formal protocol amendments. Amendments will be submitted by the coordinating centre to the relevant ethics committees for approval prior to implementation, except where immediate changes are required to eliminate an urgent hazard to participants. Approved amendments will be communicated to all participating sites with updated study documents and, where necessary, retraining of site staff. Trial registry records will be updated in a timely manner. Participants will be informed of changes that may affect their willingness to continue participation, and re-consent will be obtained when required.

## Dissemination policy {8}

Trial results will be disseminated irrespective of the direction or magnitude of findings. The trial registration record will be updated and summary results will be reported in the registry in accordance with registry requirements. Findings will be submitted to peer-reviewed journals and presented at national/international conferences for healthcare professionals and researchers. Participants who wish to receive study results will be provided with a plain-language summary after completion of the primary analyses (e.g., via WeChat/telephone or routine follow-up contact), and a lay summary may be posted through institutional communication channels. All dissemination will present aggregated results only, without any participant-identifiable information, and authorship will follow International Committee of Medical Journal Editors (ICMJE) criteria.

## Discussion

This multicentre, stratified, three-arm randomised controlled trial is designed to evaluate the efficacy and safety of a structured plant-based dietary strategy for mitigating CIGT during FOLFOX chemotherapy in patients with CRC, compared with conventional dietary guidance with or without an oncology nutritional formula. In addition to repeated clinical assessments of gastrointestinal toxicity, the trial will collect inflammatory biomarkers and stool-based multi-omics data to support exploratory investigation of biological correlates of symptom burden and treatment tolerance.

Several practical and operational issues are important in executing this study. First, blinding is inherently challenging in food-based interventions. Differences in dietary content and intervention delivery may influence participant expectations and behaviour, and cross-group contamination is possible, particularly when patients seek additional dietary advice or modify intake in response to symptoms. To reduce these risks, the protocol emphasizes standardised intervention materials and contact schedules, separation of trial roles, and blinded outcome assessment wherever feasible. Second, maintaining dietary adherence during active chemotherapy is difficult, especially across transitions between inpatient chemotherapy delivery and the home-based interval. Treatment-related nausea, anorexia, oral mucositis, taste changes, and diarrhoea may lead to symptom-driven deviations from the assigned diet [6, 7]. The protocol addresses this by combining structured education with ongoing follow-up support and by quantifying adherence and dietary exposure using prespecified dietary instruments (FFQ, diet records, and plant-based diet indices), enabling interpretation of adherence and potential dose–response relationships. Third, concomitant medications and intercurrent events can substantially affect both gastrointestinal outcomes and gut microbial profiles. Antiemetics, antidiarrheals, corticosteroids, and especially antibiotics or probiotics may modify symptoms and microbiome composition [27, 28]. The protocol therefore includes prespecified restrictions for selected agents where appropriate, systematic documentation of concomitant treatments, and integration of these factors into analysis and interpretation. Lastly, the reliability of stool-based multi-omics findings depends on strict control of pre-analytical variation [29]. Harmonized SOPs for stool collection, time stamping, storage, and batch processing across sites are intended to minimise avoidable variability and improve reproducibility of downstream analyses.

This protocol has several strengths. The three-arm design uses clinically relevant comparators that reflect real-world nutritional management during chemotherapy and allows separation of effects attributable to a structured dietary pattern from those related to nutritional supplementation alone. Stratified randomisation by site and sex improves balance for key prognostic factors, and repeated measurements across the intervention window support evaluation of symptom trajectories rather than reliance on a single endpoint. The integration of clinical endpoints with inflammatory biomarkers and stool-based profiling provides an opportunity to generate mechanistic hypotheses and to identify candidate signatures associated with symptom severity or response, while the use of prespecified analytical approaches (including appropriate control for multiple testing in high-dimensional data) supports transparent interpretation.

There are several limitations to consider. Although participant blinding will be attempted using coded programmes and role separation, it may not be fully maintained in food-based interventions due to recognisable differences in dietary content and delivery. The follow-up period is limited to six weeks and may not capture longer-term effects beyond the planned window. Multi-omics analyses are exploratory and high-dimensional; findings will require cautious interpretation and independent validation. Generalizability may also be constrained by eligibility criteria and recruitment from two hospitals in one region, which may under-represent older patients, those with severe malnutrition, or patients receiving alternative chemotherapy regimens.

If the intervention demonstrates clinically meaningful reductions in CIGT with acceptable safety, the results will support the use of a structured plant-based dietary strategy as an adjunct to standard supportive care during FOLFOX chemotherapy and inform scalable implementation pathways in routine oncology practice. Even if effects are modest, the trial will provide valuable feasibility information on adherence, retention, specimen completeness, and operational barriers that can guide refinement of dietary delivery models and the design of larger, longer-term confirmatory trials.

## Trial status

Recruitment began on 10 January 2026. As of 23 January 2026, 7 participants have been enrolled. The current protocol version is v3.0 (dated 16 January 2026). Recruitment is expected to be completed in December 2026.

## Supplementary Information

Supplementary Material 1.

Supplementary Material 2.

## Data Availability

No baseline or pilot data are reported in this protocol. Access to the final trial dataset will be restricted to the designated analysts (HRS and WP) under the oversight of the principal investigator. De-identified individual participant data will not be shared, in accordance with the ethics approval and institutional data protection policies.

## Abbreviations

CRC: Colorectal cancer
CIGT: Chemotherapy-induced gastrointestinal toxicity
ChiCTR: Chinese Clinical Trial Registry
NSFC: National Natural Science Foundation of China
KPS: Karnofsky Performance Status
FOLFOX: Oxaliplatin, leucovorin, and 5-fluorouracil
SPIRIT: Standard Protocol Items: Recommendations for Interventional Trials
NSAIDs: Nonsteroidal anti-inflammatory drugs
ID: Identifier(s)
FFQ: Food frequency questionnaire
CTCAE: Common Terminology Criteria for Adverse Events
IL-6: Interleukin-6
CRP: C-reactive protein
QC: Quality control
MS/MS: Tandem mass spectrometry
PDI: Plant-based Diet Index
hPDI: Healthful Plant-based Diet Index
uPDI: Unhealthful Plant-based Diet Index
BIA: Bioelectrical Impedance Analysis
TP: Total protein
ALB: Albumin
Hb: Haemoglobin
Fe: Iron
Mg: Magnesium
Ca: Total calcium
ALT: Alanine aminotransferase
AST: Aspartate aminotransferase
Cr: Creatinine
eGFR: Estimated glomerular filtration rate
TC: Total cholesterol
TG: Triglycerides
LDL-C: Low-density lipoprotein cholesterol
HDL-C: High-density lipoprotein cholesterol
WBC: White blood cell count
TLC: Total lymphocyte count
CEA: Carcinoembryonic antigen
CA19-9: Carbohydrate antigen 19–9
PFS-12: Piper Fatigue Scale-12
HADS: Hospital Anxiety and Depression Scale
IPAQ-C: International Physical Activity Questionnaire-Chinese version (short form)
SOPs: Standard Operating Procedures
SAP: Statistical analysis plan
SD: Standard deviation
FDR: False discovery rate
ITT: Intention-to-treat
TMG: Trial Management Group
DMC: Data Monitoring Committee
ICMJE: International Committee of Medical Journal Editors
