# Supplementary material for: Efficacy of a plant-based diet on FOLFOX chemotherapy-induced gastrointestinal toxicity in patients with colorectal cancer: study protocol for a multicentre, stratified, randomised controlled trial
Source: Trials. 2026 Mar 3;27:276. doi: 10.1186/s13063-026-09573-y (PMC13063931; doi:10.1186/s13063-026-09573-y)
Supplement: Supplementary file 2 — Supplementary Material 2. [file 13063_2026_9573_MOESM2_ESM.doc]

**Informed Consent Form**

**(Template for research projects involving pharmaceuticals and medical devices or other interventions)**

**Subject Information**

**(The italicized portions in parentheses should be filled in according to the specific requirements of the study)**

Dear :

You are invited to participate in a study investigating the effects of plant-based dietary intervention on gastrointestinal toxicity and gut microbiota in colorectal cancer patients undergoing FOLFOX chemotherapy (a regimen consisting of oxaliplatin, calcium folinate, and fluorouracil). The principal investigator is Dr. Wei Peng and his team. This research is conducted by West China Fourth Hospital of Sichuan University, and the project will apply for the National Natural Science Foundation of China for young researchers.This information sheet is provided to help you decide whether or not to participate in this scientific research. Please read it carefully, and if you have any questions, feel free to ask the researchers responsible for the study.

Your participation in this study is voluntary. This research has been approved by the Medical Ethics Committee of West China Fourth Hospital of Sichuan University.

**1. Why are we inviting you to participate in this study?**

Colorectal cancer is a common malignancy, and the FOLFOX chemotherapy regimen often causes gastrointestinal toxicity, which affects patients' quality of life and treatment outcomes. Currently, both domestic and international studies suggest that plant-based diets may have benefits for various chronic diseases, including anti-inflammatory effects and improvements in the structure and abundance of gut microbiota. However, the impact of plant-based diets on FOLFOX-induced gastrointestinal toxicity and gut microbiota in colorectal cancer patients remains limited. Therefore, conducting this study aims to provide scientific evidence for dietary management of patients and offer new methods and strategies to alleviate gastrointestinal toxicity.

**2.** **Why is this study being conducted?**

The purpose of this study is to develop a scientifically sound and effective plant-based dietary intervention plan, and to verify the safety, feasibility, and effectiveness of the intervention.

**3.** **How many people will participate in this study?**

This study will be conducted at multiple research centers, including West China Fourth Hospital of Sichuan University and Chengdu Seventh People’s Hospital. It is expected that 108 participants will voluntarily take part in the study.

**4.** **How will the study be conducted?**

Before selection for the study, researchers will collect and record your personal information (such as name, age, height, weight, diagnosis, and phone number) and conduct screenings, including body mass index, communication ability, medication use, and complications. Once your eligibility is confirmed, you will be asked to sign an informed consent form. A study number will be assigned to you, and an individual file will be created. You may be randomly assigned to one of three treatment groups: the standard diet group, the plant-based diet group, or the cancer-specific nutritional formula group. Prior to the dietary intervention (week 0) and at weeks 2 and 4 following the intervention, 15 grams of stool will be collected on three occasions for gut microbiota and metabolic analysis. Additionally, you will attend three face-to-face follow-up visits (at weeks 0, 2, and 4), each lasting approximately 15-20 minutes. All personal data and stool samples will be used solely for this study.

**5.** **How long will this study last?**

The study will last for 6 weeks, comprising a 2-week adjustment period and a 4-week intervention phase. A follow-up phone call will take place in the second week following the conclusion of the study.

**6.** **What information and biological samples will be collected in the study?**

The study will collect basic information, grade gastrointestinal toxicity reactions, and assess quality of life.

The biological samples include:

(1) Blood samples: These will be used to measure levels of inflammatory biomarkers (CRP, TNF-α, IL-6, IL-8, IL-10), as well as biochemical, metabolic, immune, and tumor markers. Blood samples will be collected as part of routine medical checks, with reports provided. The cost of collection will be borne by the patient, although the research team will offer a cash subsidy of 200 yuan;

(2) Stool samples: These will be collected for research purposes. Researchers will collect 15g of stool and send it to the Clinical Trials Center at West China Fourth Hospital of Sichuan University for storage. The cost of stool testing will be covered by the research project, and no test report will be provided.

**7. What are the risks of participating in this study?**

All of your information will be kept confidential. Sample collection will be performed under strict aseptic conditions, and stool collection will not cause any pain. Potential risks associated with the plant-based dietary intervention include weight loss, hypoglycemia, and food allergies. However, please be assured that these risks will be thoroughly assessed and mitigated prior to the intervention to ensure they do not interfere with your regular treatment.If you experience any discomfort or unforeseen circumstances during the study, regardless of whether they are related to the research, please notify the researcher (Dr. Wei Peng: 15928592228) immediately. She will evaluate the situation and provide appropriate medical or other interventions. Signing this informed consent form does not waive your right to seek compensation.

During the study, you will be required to attend follow-up visits at West China Fourth Hospital of Sichuan University or Chengdu Seventh People’s Hospital. These visits may take some of your time and could cause inconvenience.

**8. What are the potential benefits of participating in this study?**

Direct benefits: If you agree to participate in this study, you may experience direct medical benefits, such as reduced gastrointestinal toxicity following chemotherapy, improved quality of life, and smoother chemotherapy progression.

Potential benefits: This study may enhance the effectiveness of your chemotherapy, promote recovery, or slow disease progression. Your participation will contribute to the collection of data on dietary interventions for chemotherapy-induced toxicity, providing valuable evidence for the treatment of colorectal cancer and chemotherapy-related toxic reactions. These research findings have significant clinical and social value in alleviating the suffering of cancer patients and improving their quality of life.

**9.** **What are the alternative treatment options?**

If you decide not to participate in the study, you may decline without affecting your regular chemotherapy. The alternative option is the standard diet.

**10.** **How will the research results be used and how is personal privacy protected?**

If you decide to participate in this study, your participation and personal information will remain confidential. Your stool samples will be labeled with a research identification number rather than your name. Your identity will not be disclosed to anyone outside of the research team unless you grant permission. All research staff and institutions involved are required to maintain the confidentiality of your identity. Your records will be stored in a locked file cabinet, accessible only to the researchers. To ensure the research is conducted in accordance with regulations, government authorities or members of the ethics review committee may access your personal data in the research institution if necessary. When the research results are published, no personal information will be disclosed.

**11.** **What are the costs of the products and related tests used in the study?**

The food required for the study: Shelf-stable foods will be provided by the research team, while fresh vegetables and meats, which need to be purchased daily to ensure freshness, should be bought by you. The research team will compensate you with a health gift worth 500 yuan.

The cost of blood tests is part of your routine check-ups, which you will need to pay for.

The cost of stool testing is for non-routine exams, and this expense will be covered by the research project.

**12.** **Is there any compensation for participating in the study?**

Yes, there is compensation for participating in this study:

(1) You will receive professional dietary guidance, a health gift, and 200 yuan in cash (approximately 1,200 yuan per person).

(2) Free stool testing and gut microbiota metagenomic sequencing (approximately 2,500 yuan per person).

(3) The study may help reduce gastrointestinal toxicity and inflammatory responses after chemotherapy, as well as improve your nutritional status and quality of life.

**13.** **What compensation/compensation is available in case of injury?**

If any injury occurs as a result of participating in this study, you will be provided with free treatment at West China Fourth Hospital of Sichuan University, or compensation will be provided in accordance with relevant Chinese laws.

**14.** **What are your rights?**

Throughout the entire study, your participation is voluntary. If you decide not to participate, it will not affect your access to other treatments that you are entitled to. If you choose to participate, you will be asked to sign this written informed consent form. You have the right to withdraw from the study at any stage without facing discrimination or unfair treatment, and your rights will not be affected.

**15.** **What precautions should be taken?**

As a participant, you are required to provide accurate information regarding your medical history and current physical condition. You should inform the researchers of any discomfort you experience during the study. You must not take any restricted medications, foods, or other substances. Additionally, you should inform the researchers if you have recently participated in or are currently involved in any other studies.

**16.** **How can you contact your researcher?**

If any important new information arises during the study that may affect your willingness to continue participation, the researcher will inform you promptly. If you have any questions about your study data or wish to learn about the findings after the study concludes, you may ask any questions at any time and receive appropriate answers. Please contact Dr. Wei Peng at 15928592228 .

**Informed Consent Signature Page**

Informed Consent Statement:

I have been informed of the purpose, background, process, risks, and benefits of this study.

I have had sufficient time and opportunity to ask questions, and I am satisfied with the answers provided.

I have also been informed of whom to contact if I have questions, difficulties, concerns, suggestions regarding the study, or if I wish to obtain further information or assist with the study.

I have read this informed consent form.

My participation in this study is voluntary.

I have been informed that I can choose not to participate in this study or withdraw at any time by notifying the researcher without facing discrimination or retaliation, and my rights will not be affected.

If I require additional interventions, fail to comply with the study plan, or experience any research-related injury or other issues, the researcher may terminate my participation in this study.

I will receive a signed copy of this informed consent form, which includes signatures from both myself and the researcher.

Participant’s Signature: Date:＿ ＿ ＿ ＿Year＿ ＿Month＿ ＿Day

Participant’s Contact Number: Mobile Number:

Legal Representative’s Signature *(if applicable)*: Date:＿ ＿ ＿ ＿Year＿ ＿Month＿ ＿Day Legal Representative’s Contact Number: Mobile Number:

*(Note: If the participant cannot read, a witnes’s signature is required. If the participant lacks legal capacity, the signature of a legal representative is required.)*

I have accurately informed the participant of this document. He/She has read the informed consent form thoroughly and had the opportunity to ask questions.

Researcher’s Signature: Date:＿ ＿ ＿ ＿Year＿ ＿Month＿ ＿Day Researcher’s Work Phone: 　　 Mobile Number:

Office Phone of the Medical Ethics Committee of West China Fourth Hospital, Sichuan University: 028-85501599
